# Retroperitoneal Fat Necrosis in Response to an Episode of Acute Pancreatitis

**DOI:** 10.1155/2021/1051077

**Published:** 2021-09-23

**Authors:** M. B. Gilani, T. Akcan, M. Peterson, A. Zahid

**Affiliations:** ^1^Department of Internal Medicine, Marshfield Clinic Health System—Marshfield, Marshfield, WI 54449, USA; ^2^Department of Hospital Medicine, Marshfield Clinic Health System—Marshfield, Marshfield, WI 54449, USA

## Abstract

Acute pancreatitis can result in fat necrosis, typically occurring in the peripancreatic region within weeks to months, and it generally appears as a low attenuation collection, with minimal heterogeneity. There are no specific imaging features that can diagnose retroperitoneal fat necrosis which may imitate other entities including certain malignancies, which may lead to invasive studies for diagnosis. Herein, we present a case of extensive retroperitoneal fat necrosis beyond the peripancreatic region that developed 10 days after an episode of acute pancreatitis.

## 1. Introduction

Retroperitoneal fat necrosis can result as a sequela of pancreatitis due to the release of lipolytic enzymes during inflammatory processes, and its presentation varies [1]. On contrast-enhanced computerized tomography (CECT) scan, retroperitoneal fat necrosis generally appears as a low attenuation collection, with minimal heterogeneity which may imitate certain malignancies, such as carcinomatosis and liposarcoma, which may lead to further invasive studies for evaluation [1, 2]. Prior data show the interval between episodes of pancreatitis and the appearance of retroperitoneal fat necrosis varies which is generally from weeks to months rarely in days [3]. We report a case of extensive retroperitoneal fat necrosis that developed within 10 days after an episode of acute pancreatitis.

## 2. Case Presentation

**Figure 1:**
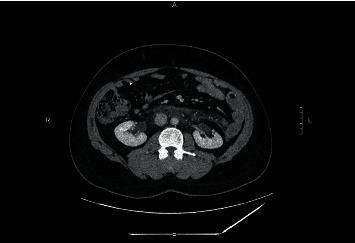
CT imaging of chest shows retroperitoneal stranding and edema adjacent to the transverse portion of duodenum and posterior to the peripancreatic head at day 1.

A 23-year-old man with a past medical history of pilocytic astrocytoma and obstructive hydrocephalus requiring a ventriculoperitoneal (VP) shunt presented with right-sided constant, intense abdominal pain that was ranked as a 10 out of 10 in intensity one day after binge drinking alcohol. The pain was mainly located on the right side of the abdomen that radiated to the back and associated with nausea and 6–8 episodes of vomiting small amounts of yellowish fluid. Initial labs showed an abnormal white blood cell (WBC) count of 18,000 per microliter and an elevated lipase level of 1,260 units per liter; the remaining labs, including comprehensive metabolic panel (CMP) and urinalysis, were in the normal range. A computed tomography (CT) scan of his chest showed retroperitoneal stranding and edema adjacent to the transverse portion of duodenum and posterior to the peripancreatic head (Figure 1). The patient was diagnosed with acute pancreatitis most likely secondary to binge drinking and was not given anything by mouth. His symptoms were managed with intravenous (IV) hydromorphone and fluids. On day three postpresentation, his pain started to improve, and he was transitioned from IV hydromorphone to oral tramadol as needed. On day five, the patient was started on a liquid diet that was slowly advanced as tolerated. The patient was discharged pain-free on day six.

**Figure 2:**
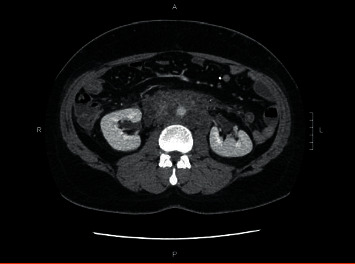
CT imaging of the abdomen and pelvis reveals large retroperitoneal process with dense infiltrative tissue extending along the aorta at day 9.

**Figure 3:**
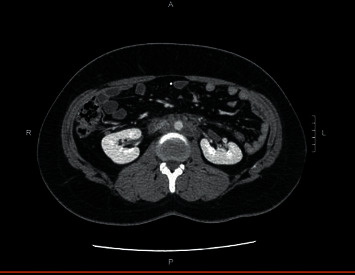
CT imaging of the abdomen and pelvis performed at four weeks shows improvement in retroperitoneal mass appearance.

**Figure 4:**
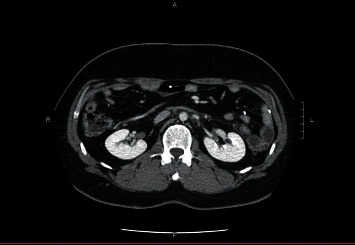
CT imaging of the abdomen and pelvis reveals significant improvement in retroperitoneal mass appearance at 12 weeks.

On day nine postpresentation, the patient returned with lower back and groin pain which was 8 out of 10 in intensity. Lab work-up was repeated and showed leukocytosis with white blood cell (WBC) count of 18,500 per microliter and elevated levels of C-reactive protein (CRP) of 22.6 mg/dL and procalcitonin of 2.34 ng/mL. His CMP and lipase activity were within the normal range. A follow-up CT of the abdomen and pelvis with contrast was done and revealed a new, large retroperitoneal process with dense infiltrative tissue extending along the aorta starting just below the origin of the superior mesenteric artery (SMA) and extending to the bifurcation measuring about 10.8 × 9.3 × 5.2 cm (Figure 2). Wispy tissue extended up along the pancreas and down the anterior pararenal spaces as well as along the iliac vessels. The retroperitoneal tissue exerted mass effect narrowing the inferior vena cava (IVC) just inferior to left renal vein and uplifted the aorta anteriorly off the spine. The pancreas enhanced normally with no pancreatic duct dilatation. Possible differentials were inflammatory sequelae from recent acute pancreatitis and retroperitoneal fibrosis, but due to the atypical distribution of inflammation, a gastroenterologist was consulted who recommended conservative management including IV hydration and pain control. On day 10, the patient spiked a fever and was empirically started on a piperacillin/tazobactam (Zosyn®) regimen while an infectious disease (ID) specialist was consulted. The ID specialist recommended a tissue biopsy of the retroperitoneal process and further laboratory work-up for infection. A CT-guided aspiration of the retroperitoneal lesion was done and histology testing performed specifically potassium hydroxide preparation (KOH) smear, fungal culture, acid-fast bacillus (AFB) smear and culture, and aerobic and anaerobic cultures with subsequent Gram stain. Other work-up for infections included blastomycosis urinary antigen, interferon gamma release assay, blood cultures, and urinalysis. Results were either inconclusive or negative. Zosyn was continued for a total of seven days and discontinued upon receipt of infectious disease work-up results. The patient was discharged from the hospital and additional CT imaging of the abdomen and pelvis was performed at four (Figure 3) and 12 weeks (Figure 4) that showed significant improvement in retroperitoneal mass appearance.

## 3. Discussion

Fat necrosis is a known complication of acute pancreatitis [2, 3] that can be detected with contrast-enhanced computerized tomography (CECT) [4]. Conservative management of sterile fat necrosis is an accepted practice and is associated with a low (12%) mortality [5]. According to Smith et al., “The distribution of fat necrosis is typically peripancreatic, with extensions to the origin of the mesentery of the small intestine, the transverse mesocolon, and the omentum” [2].

The initial retroperitoneal stranding seen in our patient was consistent with a typical distribution; however, subsequent CECT findings of extensive fat necrosis have, to our knowledge, not previously been reported in association with pancreatitis. In addition, the development of retroperitoneal fat necrosis occurred within 10 days of acute pancreatitis, which varied from the normal time interval of weeks to several months [3]. The patient in this report showed marked improvement with CECT at 4 and 12 weeks postpancreatitis, in contrast to a similar case which showed persistence of peritoneal deposits after six months [2].

Consultation with oncology led to a negative work-up for carcinomatosis, based in part of the patient's age and the rapid progression of the mass. Due to the consistent decrease in size of the fat necrosis over time, malignancies were not considered as a potential diagnosis [6]. A literature review found one case of extensive retroperitoneal fat necrosis in a case of renal cell carcinoma [7].

Sterile necrosis has been historically managed conservatively to prevent the introduction of an infectious agent into the body [5]. Retroperitoneal fat necrosis secondary to acute pancreatitis is currently not preventable; this case demonstrates a clinically favorable outcome with conservative management and monitoring with CECT.

## Data Availability

No data were used to support this study.
